# Distinct Signatures of Tumor-Associated Microbiota and Metabolome in Low-Grade vs. High-Grade Dysplastic Colon Polyps: Inference of Their Role in Tumor Initiation and Progression

**DOI:** 10.3390/cancers15123065

**Published:** 2023-06-06

**Authors:** Michela Giulia Clavenna, Marta La Vecchia, Marika Sculco, Soni Joseph, Elettra Barberis, Elia Amede, Marta Mellai, Silvia Brossa, Giulia Borgonovi, Pietro Occhipinti, Renzo Boldorini, Elisa Robotti, Barbara Azzimonti, Elisa Bona, Edoardo Pasolli, Daniela Ferrante, Marcello Manfredi, Anna Aspesi, Irma Dianzani

**Affiliations:** 1 Department of Health Sciences, Università del Piemonte Orientale, 28100 Novara, Italy; michela.clavenna@uniupo.it (M.G.C.); marta.lavecchia@uniupo.it (M.L.V.); marika.sculco@uniupo.it (M.S.); soni.joseph@uniupo.it (S.J.); marta.mellai@uniupo.it (M.M.); renzo.boldorini@med.uniupo.it (R.B.); barbara.azzimonti@med.uniupo.it (B.A.); anna.aspesi@med.uniupo.it (A.A.); 2 Department of Translational Medicine, Università del Piemonte Orientale, 28100 Novara, Italy; elettra.barberis@uniupo.it (E.B.); elia.amede@uniupo.it (E.A.); daniela.ferrante@med.uniupo.it (D.F.); marcello.manfredi@uniupo.it (M.M.); 3 Center for Translational Research on Autoimmune and Allergic Diseases, University of Piemonte Orientale, 28100 Novara, Italy; 4 Department of Gastroenterology, “Maggiore della Carità” Hospital, 28100 Novara, Italy; 5 Department of Sciences and Technological Innovation, Università del Piemonte Orientale, 15121 Alessandria, Italy; elisa.robotti@uniupo.it; 6 Department for Sustainable Development and Ecological Transition, Università del Piemonte Orientale, 13100 Vercelli, Italy; elisa.bona@uniupo.it; 7 Department of Agricultural Sciences, University of Naples Federico II, 80055 Portici, Italy; edoardo.pasolli@unina.it; 8 Task Force on Microbiome Studies, University of Naples Federico II, 80055 Portici, Italy

**Keywords:** mucosa-associated microbiota, lumen-associated microbiota, microbiota-derived metabolites, gut, colon polyp, colorectal cancer, driver bacteria, passenger bacteria

## Abstract

**Simple Summary:**

A growing body of research has shown the connection between gut microbiota and colorectal cancer. However, most studies analyze fecal microbiota, which do not reliably represent the bacterial populations associated with colon mucosa. We analyzed the microbiota and metabolome directly collected from the surface of colon polyps, and showed different bacterial and metabolite signatures that discriminate between patients with low- and high-grade dysplastic polyps. We identified bacterial genera and species that are enriched in the early stages of tumor development and may act as drivers of carcinogenesis. Moreover, we revealed that differences in metabolite profiles accompanied the changes in bacterial composition associated with tumor stage, and that gut bacteria are involved in the production and consumption of significantly altered metabolites. Our findings pave the way for future mechanistic investigations to elucidate the role of specific bacteria in colon carcinogenesis and to design preventative measures based on microbiota modulation.

**Abstract:**

According to the driver–passenger model for colorectal cancer (CRC), the tumor-associated microbiota is a dynamic ecosystem of bacterial species where bacteria with carcinogenic features linked to CRC initiation are defined as “drivers”, while opportunistic bacteria colonizing more advanced tumor stages are known as “passengers”. We reasoned that also gut microbiota-associated metabolites may be differentially enriched according to tumor stage, and be potential determinants of CRC development. Thus, we characterized the mucosa- and lumen-associated microbiota (MAM and LAM, respectively) and mucosa-associated metabolites in low- vs. high-grade dysplastic colon polyps from 78 patients. We show that MAM, obtained with a new biopsy-preserving approach, and LAM differ in composition and α/β-diversity. By stratifying patients for polyp histology, we found that bacteria proposed as passengers by previous studies colonized high-grade dysplastic adenomas, whereas driver taxa were enriched in low-grade polyps. Furthermore, we report altered “mucosa-associated metabolite” levels in low- vs. high-grade groups. Integrated microbiota-metabolome analysis suggests the involvement of the gut microbiota in the production and consumption of these metabolites. Altogether, our findings support the involvement of bacterial species and associated metabolites in CRC mucosal homeostasis in a tumor-stage-specific manner. These distinct signatures may be used to distinguish low-grade from high-grade dysplastic polyps.

## 1. Introduction

Colorectal cancer (CRC) is among the most prevalent cancers worldwide, with more than 1.9 million cases and 935,000 deaths in 2020 [1]. Its multifactorial etiology includes genetic, environmental, and life-style factors. In most cases, CRC develops from adenomatous polyps, which show different grades of dysplasia during tumor progression.

Mounting evidence indicates that changes in the gut microbiota play an important role in colon carcinogenesis [2].

The bacterial driver–passenger model for CRC proposes that bacterial species have distinct temporal associations with colorectal tissues according to their role in CRC pathogenesis [3,4,5]. Driver bacteria are found in the initial stages of carcinogenesis and are, therefore, thought to play a role in CRC initiation by different mechanisms: production of genotoxic substances, disruption of the function of tumor suppressor proteins, such as E-cadherin, production of metabolites that increase the proliferation of enterocytes and opportunistic microbial pathogens, and induction and maintenance of the inflammatory process [3,4,6]. Passenger bacteria are instead opportunistic pathogens involved in CRC progression [3,7]. One of the most studied driver bacteria is the enterotoxigenic strain of *Bacteroides fragilis* (ETBF) that can facilitate the initiation of pre-malignant lesions through the release of enterotoxins (BFTs) [3]. The epithelial response to *B. fragilis* toxins induces E-cadherin cleavage, resulting in enhanced barrier permeability, Wnt/β-catenin, and NF-κB signaling [8]. Importantly, tumor-susceptible mice (Apc^Min/+^) colonized by ETBF strains are used as a model of microbial-induced colon tumorigenesis [9]. Another example of driver bacteria is represented by *Escherichia coli* strains harboring the polyketide synthase (PKS) genomic island and capable of synthesizing the genotoxic virulence factor colibactin, which induces double-strand DNA breaks [10].

Given that the tumor microenvironment (TME) changes during the oncogenic process, pathogenic driver bacteria can be numerically overwhelmed and gradually replaced by passenger bacteria that acquire a growth advantage in the tumor context. These opportunistic pathogens, which otherwise would not be able to colonize healthy colorectal tissues, exploit the altered metabolism of the tumor colonocytes to proliferate [3,4]. Passenger bacteria may also be actively involved in cancer progression, though their relevance is still unclear [3,4,11].

Another mechanism through which microbiota can influence CRC development is the production of various metabolites [12,13]. It is, in fact, widely accepted that microbiota-derived metabolites play a crucial role in host physiology and disease development, and that their abundances may vary according to tumor stage. Among the most heavily studied microbiota-derived metabolites in CRC, are short-chain fatty acids (SCFAs) and bile acids (BAs) [14]. In particular, deoxycholic acid, which is a secondary bile acid, can cause inflammation and promote intestinal tumorigenesis in Apc^Min/+^ mice [15]. Bile acid is involved in tumor progression through the activation of the NF-κB pathway, which promotes cell growth and survival [16]. Moreover, gut-associated metabolites may directly alter the gut microbiota composition by promoting the proliferation of specific bacteria, in particular that of passengers [17].

A number of studies have performed integrated analyses on lumen-associated microbiota (LAM) and metabolome from fecal or serum samples to characterize the role of bacteria and associated metabolites in the pathogenesis of CRC [18,19,20]. However, data are scant on gut mucosa-associated microbiota (MAM) and mucosa-associated metabolome.

The emerging consensus is that the composition of gut MAM differs from that of LAM, with only a few species being present in both compartments [21,22,23,24,25].

Based on the aforementioned evidence, the aim of this study was to characterize the temporal association of MAM and their metabolites with low-grade vs. high-grade dysplastic colon polyps—which represent two distinct stages of the adenoma–carcinoma sequence—and to ascertain their role in neoplastic development. For this purpose, we have devised a new technique that allows the collection of bacteria and metabolites from the adenoma’s surface without jeopardizing tissue integrity.

The analysis of MAM and mucosal-associated metabolome, according to the histological classification of colon polyps, reveals the preponderance of potential driver bacteria in low-grade dysplastic polyps, while potential passenger bacteria are enriched in high-grade dysplastic ones. The bacteria classification is based on previous reports, where candidate drivers or passengers were proposed [3,4], albeit no functional experiments were performed to show that these bacteria play a direct role in malignancy. We also report differences in the metabolite relative abundances between the two study groups, suggesting that these signatures may be used to distinguish colon polyps according to histology. Finally, integrated analysis of MAM and metabolites shows either positive or negative correlations between enriched bacteria and specific classes of metabolites, supporting the involvement of the gut microbiota in the production and consumption of these metabolites.

## 2. Materials and Methods

### 2.1. Patients Enrollment

Patients (*n* = 78, males = 45; females = 33) were recruited before colonoscopy at the Gastroenterology Unit of Maggiore della Carità University Hospital (Novara, Italy). All the patients undergoing colonoscopy signed an informed consent form. After colonoscopy, only patients with polyps larger than 10 mm, older than 18 years were included in this study. The other exclusion criteria were prebiotic, probiotic or antibiotic consumption within one month before fecal sample (LAM) collection. Previous gastro-intestinal conditions that could modify the gut microbiota were evaluated, including diverticula, cholecystectomy and previous polyp occurrence and reported in Table 1 and Table S1. All the patients used laxatives before colonoscopy, as required by the procedure. A team that includes 13 medical operators of the same Unit, all using the same working procedures, performed all the colonoscopies, and a single nurse collected all the microbiome and the metabolome samples.

### 2.2. Sample Collection

To collect MAM and associated metabolites, e-NAT™ (COPAN, Brescia, Italy) swabs or dry swabs, respectively, were used to gently brush the polyp surfaces without compromising their tissue integrity. Samples were stored at −80 °C until 16S rRNA tag sequencing and metabolite extraction. Bowel preparation could alter gut microbiota and metabolome composition [26]. Nagata and colleagues in 2019 showed that after 14 days, both microbiota and metabolome are completely restored [27]. Therefore, for LAM analyses, fecal samples were collected from the patients 14 days after colonoscopy, aliquoted and stored at −80 °C until microbial DNA isolation and sequencing.

Patient nutritional habits were evaluated with the validated European Prospective Investigation into Cancer and nutrition (EPIC) questionnaire on nutrition [28]. The questionnaire is composed of 16 categories and questions about 266 different items, including simple foods and recipes, to understand the food frequency intake. The questionnaire was completed online and analyzed, and the intake frequency was transformed in grams/day. We analyzed and compared the consumption of the most important nutrients in low- vs. high-grade dysplasia groups (Table S2).

### 2.3. Histology

After removal, polyps were included in neutral buffered formalin for at least 24 h and then included in paraffin. Sections were cut at 4-μm thickness and stained using hematoxylin-eosin. Polyps were oriented using a stereo microscope and cut alongside the major axis, identifying, if possible, the base implant. All polyps (i.e., tubular, villous, tubulovillous, sessile-serrated) were evaluated by an expert pathologist (R.B.) at the University Hospital Pathology Unit in Novara, Italy. Patients with low-grade dysplastic adenomas were included in the “low-grade” group, while patients with high-grade dysplastic adenomas were included in the “high-grade” group (Table 1).

### 2.4. MAM and LAM Analyses

Microbial DNA for MAM analyses were extracted from e-NAT™ swabs with QIAamp^®^ DNA Microbiome kit (Qiagen, Hilden, Germany), according to the manufacturer’s instructions.

LAM analyses were performed on microbial DNA extracted from fecal samples using QIAamp^®^ PowerFecal^®^ Pro DNA kit (Qiagen, Hilden, Germany), according to the manufacturer’s instructions. The yield and quality of microbial DNA was determined using a NanoDrop™ 2000 spectrophotometer (Thermo Fisher Scientific Inc., Waltham, MA, USA). The quantity was assessed with Invitrogen™ Qubit™ 1X dsDNA HS Assay Kit (Invitrogen Co., Thermo Fisher Scientific Inc.) using a Qubit 4 fluorometer (Invitrogen).

To avoid contaminations, microbial DNA extraction for MAM and LAM was performed in sterile conditions, using a laminar flow cabinet and sterile reagents and materials. E-NAT™ swabs not brushed on any tissue were used as negative controls for microbial DNA extraction and 16S rRNA sequencing.

MAM and LAM samples were subjected to 16S rRNA amplicon sequencing analysis using Microbiota Solution B Kit, a next-generation sequencing (NGS) in vitro molecular test, CE-IVD marked (Arrow Diagnostics Srl, Genoa, Italy). Polymerase chain reaction (PCR) amplification of the V3-V4-V6 hypervariable regions of bacterial 16S rRNA was obtained by using the patented degenerate primer sets within the Arrow Microbiota Solution B kit (cod. AD-002.024), according to the manufacturer’s instructions. PCR products were purified using Agencourt AMPure XP magnetic beads (Beckman Coulter Inc., Brea, CA, USA), and indexes were added in a subsequent step. The hypervariable V3-V4-V6 regions of the bacterial 16S rRNA were amplified according to the manufacturer’s instructions.

The DNA concentration of the libraries was fluorometrically measured and samples were pooled in equimolar concentrations. The final 16S rRNA amplicon libraries were sequenced on a MiSeq Illumina^®^ sequencing platform (Illumina, San Diego, CA, USA) using a MiSeq Reagent Nano Kit v2 cartridge for a 2 × 250 paired-end sequencing.

### 2.5. Phylogenetic Investigation of Communities by Reconstruction of Unobserved States (PICRUSt)

Functional abundances were predicted using the Phylogenetic Investigation of Communities by Reconstruction of Unobserved States (PICRUSt2) software 2.0 [29]. Pathways differentially abundant between low- and high-grade dysplastic polyps were detected using the STAMP software [30]. Pathways with *p*-value < 0.05 were identified as significant after false discovery rate (FDR) correction. We consulted the MetaCyc website (https://metacyc.org/, accessed on 26 September 2022) to identify the products of each pathway which emerged from the PICRUSt analysis.

### 2.6. Mucosa-Associated Metabolome

Small molecules were extracted and analyzed as reported in our previous validated method [31]. Briefly, short chain fatty acid (SCFA) extraction from dry swabs was performed first using water and sonication and then liquid-liquid extraction with methyl tert-butyl ether (MTBE). Methanol-isopropanol-acetonitrile was then used to extract other metabolites (i.e., amino acids, sugars, long fatty acids, and medium fatty acids) from the aqueous phase. The internal standards deuterated propanoic acid (1 ppm), tridecanoic acid (0.5 ppm) and hexadecane (1 ppm) were also added. SCFAs and small molecules were analyzed by bidimensional gas chromatography mass spectrometry GCXGC/TOFMS (BT 4D, Leco Corp., St. Josef, MI, USA), as described in our previous work [31]. The samples were analyzed using both targeted and untargeted approaches. Briefly, SCFAs were quantified using a targeted analysis performed with internal standards and external calibration curves, as previously reported [31]. For the untargeted analysis, peaks with signal-to-noise (S/N) value lower than 500.0 were rejected. ChromaTOF version 5.31 was used for raw data processing and mass spectral assignment was performed by matching with NIST MS Search 2.3 libraries adding Fiehn Library. Identification of molecules was also performed using an in-house library built with commercial mix standards that contain hundreds of molecules. As the polyp mean area was different between the low- and high-grade dysplasia groups (median (IQR) 12 (10–16) mm vs. 15 (12–25) mm; *p*-value ≤ 0.05), normalization was performed by dividing the metabolites’ abundances by the value of the area of each analyzed polyp, with the limit of the type and shape of the polyps. Measurements were performed at the time of colonoscopy with graph paper. The internal standards that were spiked in each sample, were used for instrument stability monitoring and data normalization. In addition, small molecule levels from untargeted analysis were also normalized by total sum of abundances. To study a possible correlation between polyp-associated microbiota and its metabolites, the metabolome analysis was integrated with MAM using M^2^IA, an open-source web server. The hierarchical clustering heat map analysis was performed through MetaboAnalyst software 5.0 (www.metaboanalyst.ca, accessed on 17 December 2021) using the Euclidean distance as distance measure and the Ward method as clustering method. Only modulated metabolites (*p*-value < 0.05 and fold change > 1.3 or <0.769) were used. Metabolomics data are shown in Table S3.

### 2.7. Raw Sequence Processing

Raw sequences obtained from MAM and LAM DNA were processed using the software MicrobAT Suite v1.2.1 (SmartSeq srl, Novara, Italy), based on the Ribosomal Database Project (RDP) database. MicrobAT (SmartSeq s.r.l.) is a standalone software based on client/server system. Through a graphical interface developed in Java, the user can load the FASTQ files, download the raw data of the analysis and print the reports of the samples. The first step is a cleaning of the reads obtained from the FASTQ file using custom algorithms that remove the short sequences (read length < 200 nt) and sequences with a low quality (average Phred quality score [32] < 25). High-quality sequences are then aligned with the reference database, i.e., RDP database release 11-update 5 [33]. During this taxonomic assignment process, only the reads with minimum sequence length that align with reference ≥80% and similarity threshold ≥97% were associated, by the analysis system, with the species taxonomic level. Finally, the software generates absolute abundance tables and three files (OTU, taxonomy, metadata) used as input for the subsequent analyses [34,35].

Statistical analysis regarding variations within the bacterial communities was performed using MicrobiomeAnalyst software 1.0 (Comprehensive Statistical, Visual, and Meta-Analysis of Microbiome data) [36].

Firstly, a data integrity check was performed by the online software to show the information collected. Secondly, taxa having zero reads across all the samples or appearing in only one sample were removed by default. Finally, a low-count filter was applied to remove taxa containing less than 30 (LAM vs. MAM comparison) or 10 reads (low- vs. high-grade dysplasia comparison) in at least 20% of samples.

### 2.8. Statistical Analysis

Fisher’s exact test was used to compare the groups, as reported in Table 1.

Heat tree analysis was used to compare statistically significant differences between the groups, i.e., MAM vs. LAM, low- vs. high-grade dysplastic polyps. This method, performed through R metacoder package [37], uses hierarchical structure of taxonomic classifications to quantitatively (median abundance) and statistically (non-parametric Wilcoxon Rank Sum test) depict taxon differences among communities, using color and size of nodes.

To compare MAM with LAM, data were summarized using α- or β-diversity indexes. Three α-diversity metrics were used: the observed number, the Shannon index, and the Simpson index. The first index evaluates the number of unique taxa observed in each sample, considering only richness. The last two are based on not only richness but also evenness, which represents the abundance of a given microorganism. We performed α-diversity analysis using the phyloseq package [38], and results were plotted across samples and depicted as box plots for each group.

Beta diversity analysis, used to compare the different composition between the analysis groups, was calculated by Bray–Curtis distance, and the results were visualized in two plots through principal coordinate analysis (PCoA). In the plots, each point represents the entire microbiome of a single sample. The statistical significance of the differences in β-diversity between groups (MAM vs. LAM; low- vs. high-grade dysplastic polyps) was evaluated using permutational ANOVA (PERMANOVA).

Linear discriminant analysis effect size (LDA-LEfSe) was used to identify signatures at different taxonomic levels, characterizing each different group (MAM vs. LAM, low- vs. high-grade dysplastic polyps). This method estimates both statistical significance and biological consistency (effect size). Firstly, it uses the Kruskal–Wallis sum-rank test to identify taxa that are statistically different between groups. Subsequently, LEfSe applies LDA to calculate the effect size of each differentially abundant feature. Features with *p* < 0.05 and an LDA score > or <2 were considered taxa able to discriminate between the two groups. For mucosal-adherent and luminal microbiota comparison, the false discovery rate (FDR) was used to correct for multiple testing, and taxa with *p*-values < 0.05 were considered statistically significant. The *p*-values adjusted for the FDR are indicated as *q*-values.

For the correlation analyses between polyp-associated microbiota and metabolites, Spearman’s rank correlation coefficient was calculated using the M^2^IA web server with default settings.

## 3. Results

### 3.1. Characterization of LAM and MAM in Patients with Colon Polyps

Contrary to other sample collection protocols that cause the degradation of the biopsies to obtain microbiota and metabolites, we have developed a new approach that allows us to analyze the microbiota and metabolome adherent to the polyp surface without compromising the integrity of the biopsies, a key requisite to perform an accurate histological analysis.

Seventy-eight patients (45 males, 33 females) with polyps larger than 10 mm were recruited before colonoscopy at the Gastroenterology Unit of the University Hospital Maggiore della Carità in Novara, Italy. The clinical features of this study population are shown in Table 1. We did not find any statistically significant difference between low- and high-grade groups regarding previous gastro-intestinal conditions and polyp localization (Table 1). Since diet can influence the gut microbiota composition, we compared nutrient consumption between patients with low- and high-grade dysplastic polyps. We did not find any statistically significant difference in the daily consumption of fiber, lipids, red and processed meat, fruit and vegetables, as shown in Table S2.

**Figure 1:**
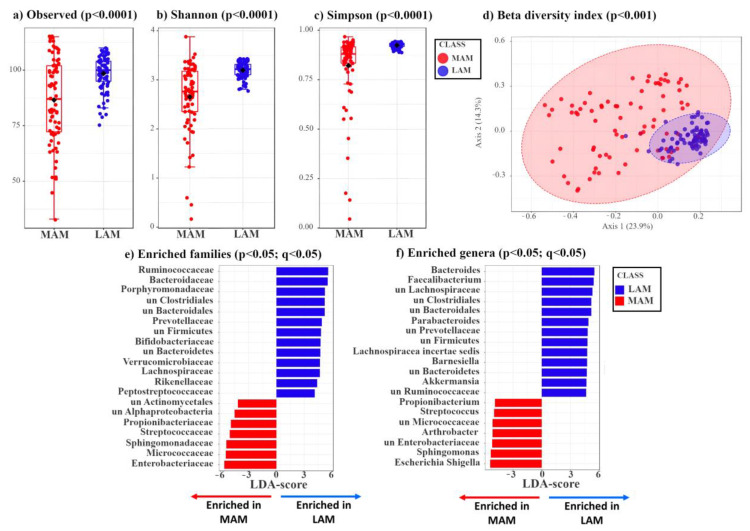
Box plots showing three different α-diversity indexes tested by Wilcoxon rank sum test. (**a**) The observed index is a richness-based measure showing the number of unique taxa present in each sample. The box plot shows the difference in observed species between MAM (red) and LAM (blue) samples (*p* = 1.17 × 10^−4^). (**b**,**c**) Shannon and Simpson indexes are based on richness (i.e., numbers of species) and evenness (i.e., abundance of microorganisms). The two different box plots show significant differences between MAM (red) and LAM (blue) samples (Shannon: *p* = 3.49 × 10^−10^, Simpson: *p* = 1.05 × 10^−11^). (**d**) β-diversity analysis shows a significant separation between MAM (red) and LAM (blue) samples (*p* < 0.001). Principal coordinates analysis (PCoA), based on Bray–Curtis distance matrix, shows the different microbial composition between the two groups. This is achieved by comparing the changes in presence/absence or abundance of thousands of species and by summarizing how “similar” or “dissimilar” they are. The X-axis explains 23.9% of the variability between samples, while the Y-axis explains 14.3%. The statistical significance of the clustering pattern was calculated using permutational ANOVA (PERMANOVA). (**e**,**f**) Linear discriminant analysis effect size (LDA-LEfSe) showing families (**e**) and genera (**f**) enriched in LAM (blue, on the right, LDA-score > 3) or MAM (red, on the left, LDA-score < −3).

MAM samples were collected by gently brushing the surface of the resected polyp with an e-NAT^TM^ swab, which allows the preservation of the nucleic acids until extraction, whereas LAM-containing specimens were isolated from feces using a standard approach (see Methods). Subsequently, MAM and LAM samples were subjected to 16S rRNA sequencing, yielding an average number of reads of 53,028.73 and 67,479.96, respectively. After applying a low-count filter to remove taxa showing less than 10 reads, we obtained 165 taxa from the MAM samples and 202 from the LAM ones. These genomic sequences were included in the BioProject MIMEC Project_Swab PRJNA783496 and MIMEC Project_Fecal PRJNA783535 available in the NCBI database https://submit.ncbi.nlm.nih.gov/subs/sra/SUB11427238/overview, accessed on 17 December 2021 and https://submit.ncbi.nlm.nih.gov/subs/sra/SUB11420448/overview, accessed on 17 December 2021, respectively. The α-diversity indexes—which include the observed number (Figure 1a), the Shannon (Figure 1b) and the Simpson (Figure 1c) indexes—show that LAM is characterized by a significantly higher mean species diversity than that of MAM (*p* < 0.05).

Next, we assessed the β-diversity indexes of MAM vs. LAM by principal coordinates analysis (PCoA) using the Bray–Curtis distance matrix. As shown in Figure 1d, LAM displays a tighter clustering compared to that of MAM. The statistical significance of the clustering pattern was confirmed by permutational ANOVA (PERMANOVA) (*p* < 0.001, Figure 1d).

Compared to MAM, LAM shows a phylum enrichment of Firmicutes (Bacillota) (51.30% vs. 39.03%), Bacteroidetes (Bacteroidota) (22.03% vs. 7.30%), and Verrucomicrobia (Verrucomicrobiota) (1.72% vs. 1.00%) (*p* < 0.05) (Figure S1a,b). Conversely, MAM displays a phylum enrichment of Proteobacteria (Pseudomonadota) (15.91% vs. 2.52%) and Actinobacteria (Actinomycetota) (11.79% vs. 6.27%) (Figure S1a,b). The two groups (MAM vs. LAM) show statistically significant differences at phylum, class, order (Figure S1c–e), and at family and genus level (Figure 1e,f).

LDA LEfSe analysis was carried out to identify the bacterial genera that were enriched in the LAM or MAM samples (Table S4). In agreement with the phylum results, all genera included in the Bacteroidetes (Bacteroidota) and Verrucomicrobia phyla and most genera included in the Firmicutes phylum (Bacillota) are significantly enriched (*p* < 0.05) in LAM compared with MAM, whereas most genera included in Proteobacteria (Pseudomonadota) and Actinobacteria (Actinomycetota) are more abundant in MAM samples (Table S4).

Overall, these data suggest that patients’ MAM differs from LAM, possibly because MAM is more related to the localized changes occurring near the polyps and more dependent of tumor stage, while LAM is representative of all bacterial species present in the gut. Thus, even though LAM has a higher number of species (α-diversity), most of these latter are shared among patients regardless of tumor stage.

### 3.2. Identification of Mucosa-Associated Bacterial Signatures Distinguishing Low-Grade from High-Grade Dysplastic Colorectal Polyps

Since bacteria in close contact with enterocytes may play an important role in colon carcinogenesis, we focused our attention on polyp-associated microbiota. In particular, we asked whether the MAM characterizing low-grade dysplastic polyps would be enriched in driver bacteria, which influence the initial stages of carcinogenesis, whereas MAM of high-grade dysplastic polyps would be enriched in passenger species.

**Figure 2:**
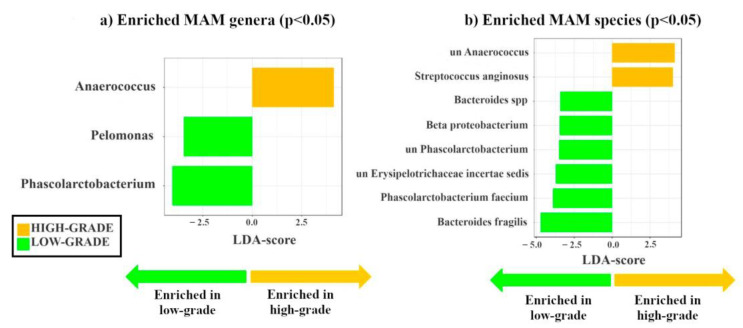
Linear discriminant analysis effect size (LDA-LEfSe) showing (**a**) bacterial genera and (**b**) species enriched in MAM high- (yellow, LDA score > 2) vs. low-grade dysplastic adenomas (green, LDA score < −2). This method incorporates statistical significance (Kruskal–Wallis) with biological consistency (effect size). The length of the bar represents a log10 transformed LDA score. This value is positive if the bacterial species is enriched in the first compared to the second group and negative if the second group shows enrichment compared to the first group. A significance level of *p* < 0.05 and an LDA score of 2 are used to determine the species best characterizing each phenotype.

By stratifying patients according to histology (low-grade vs. high-grade dysplasia), we identified two genera (*Pelomonas* and *Phascolarctobacterium*) enriched in low-grade, while the potential passenger genus *Anaerococcus* [4] was enriched in high-grade dysplastic polyps (Figure 2a). Moreover, we found the potential driver species, *Bacteroides fragilis* [3] and five other species (i.e., *Bacteroides* spp., *Beta proteobacterium,* unclassified *Phascolarctobacterium,* unclassified *Erysipelotrichaceae incertae sedis*, and *Phascolarctobacterium faecium*) enriched in low-grade dysplastic polyps (*p* < 0.05), whereas the two potential passenger species, unclassified *Anaerococcus* and *Streptococcus anginosus* [4], were enriched in high-grade dysplastic polyps (Figure 2b). Thus, as we hypothesized, known candidate driver taxa are only enriched in MAM of low-grade dysplastic polyps, while known candidate passenger taxa are only enriched in MAM of high-grade dysplastic polyps. It must be considered that candidate driver or passenger classification is based on previously suggested classifications, lacking validation by functional experiments.

Similar differences in MAM between low- and high-grade dysplastic polyps were observed by analyzing the phylogenetic heat tree (Figure S2).

It is noteworthy that the analysis of LAM also revealed different signatures for genera and species in high- and low- grade dysplastic groups (Figure S3).

### 3.3. Comparison of Mucosa-Associated Metabolome between High-Grade and Low-Grade Dysplastic Colorectal Polyps

Next, we asked whether there was an association between tumor stage and the composition of the mucosa-associated metabolome. To answer this question, we identified the metabolome adherent to the polyps through a high-throughput metabolomics approach recently described by our group [31]. Of note, metabolites in the gut can derive from bacteria, endogenous compounds, or exogenous dietary components [39,40,41].

Metabolome analysis of 59 (34 low- vs. 25 high-grade) out of 78 patients (19 samples were unavailable) uncovered 41 metabolites that allowed us to distinguish between high- and low-grade dysplastic polyps (fold change, FC > 1.3 enriched in high-grade or FC < 0.769 depleted in high-grade; *p* < 0.05). In high-grade polyps, we found a higher concentration of SCFAs, such as butyric acid (FC = 3.7; *p* < 0.05) and isobutyric acid (FC = 3.5; *p* < 0.05), lactic acid (FC = 1.9; *p* < 0.05), the nucleobase uracil (FC = 3.6; *p* < 0.01), and several amino acids, such as threonine (FC = 8.2; *p* < 0.01), serine (FC = 2.8; *p* < 0.05), α-aminobutanoic acid (FC = 5.6; *p* < 0.05). Other differentially abundant metabolites included erythronic acid (FC = 0.7; *p* < 0.05), L-threitol (FC = 1.9; *p* < 0.05), pyroglutamic acid (FC = 4.7; *p* < 0.05), and hydroquinone (FC = 2.8; *p* < 0.01) (Figures S4 and S5).

**Figure 3:**
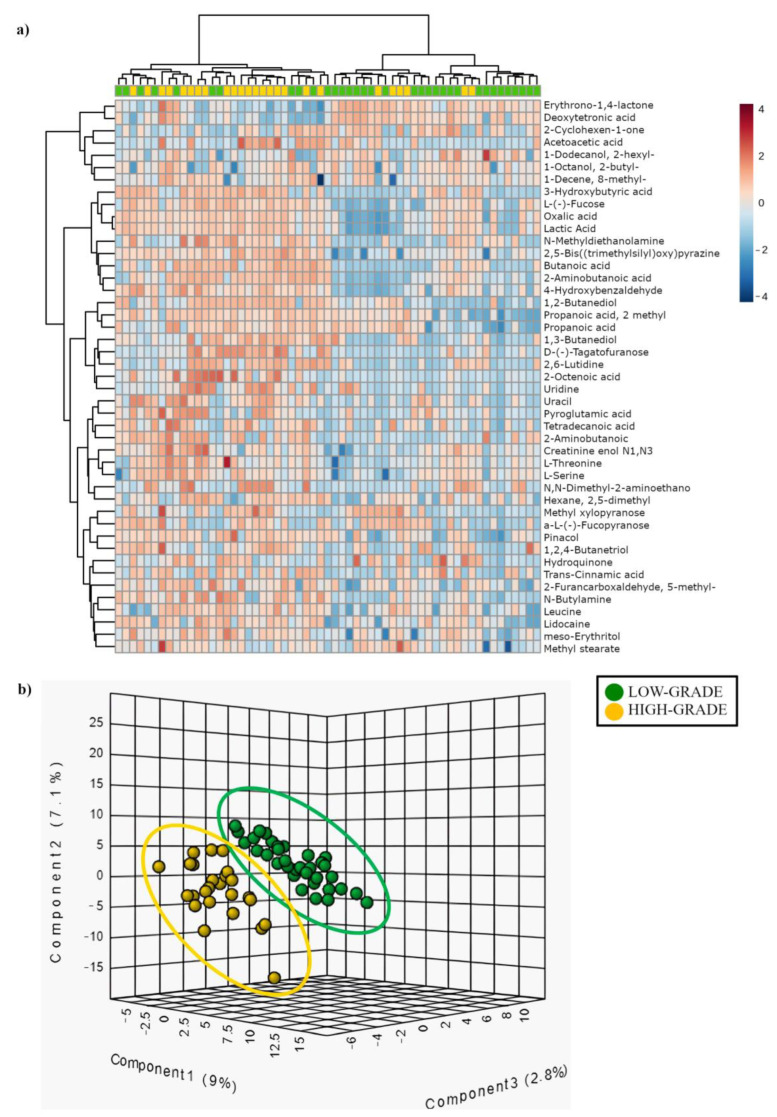
(**a**) Hierarchical clustering heat-map showing different metabolite distributions between low- (green) and high-grade (yellow) dysplastic polyps. All the metabolites listed show a statistically significant difference between low and high-grade dysplasia groups (*p* < 0.05). Higher concentrations are reported in red, while low levels are in blue (auto-scaled data). (**b**) Partial least square discriminant analysis (PLS-DA) showing different metabolite distribution between low- (green) and high-grade (yellow) dysplastic polyps.

The hierarchical clustering heat map (Figure 3a and Figure S6) shows the distribution of the metabolites that are statistically different between low- (green) and high-grade (yellow) dysplastic polyps. The partial least square discriminant analysis (PLS-DA) reported in Figure 3b shows the presence of a metabolic signature associated with low- (green) or high-grade (yellow) dysplastic polyps.

### 3.4. Phylogenetic Investigation of Communities by Reconstruction of Unobserved States (PICRUSt) in Low-Grade vs. High-Grade

PICRUSt analysis was performed to predict the metabolic function of bacteria found differently enriched in the low- or the high-grade group. PICRUSt analysis on MAM identified 17 different pathways statistically enriched in low- or high-grade dysplastic polyps (4 enriched in low-grade and 13 enriched in high-grade). We show these pathways in Table 2. We consulted the MetaCyc website (https://metacyc.org/, accessed on 26 September 2022) to identify the products of each pathway which emerged from the PICRUSt analysis. In particular, in Figure S7, we show the mixed acid fermentation pathway and superpathway of the pyrimidine ribonucleosides salvage, which were enriched in the high-grade group.

### 3.5. Integration of MAM and Polyp-Adherent Metabolome Data

In order to investigate which bacterial taxa and small molecules/metabolite classes were mainly responsible for the overall associations with the histological grade of polyps, the individual correlations between genus level, bacterial abundance profile, class level, and individual metabolite level intensity profile were analyzed using the M2IA open-source web server.

**Figure 4:**
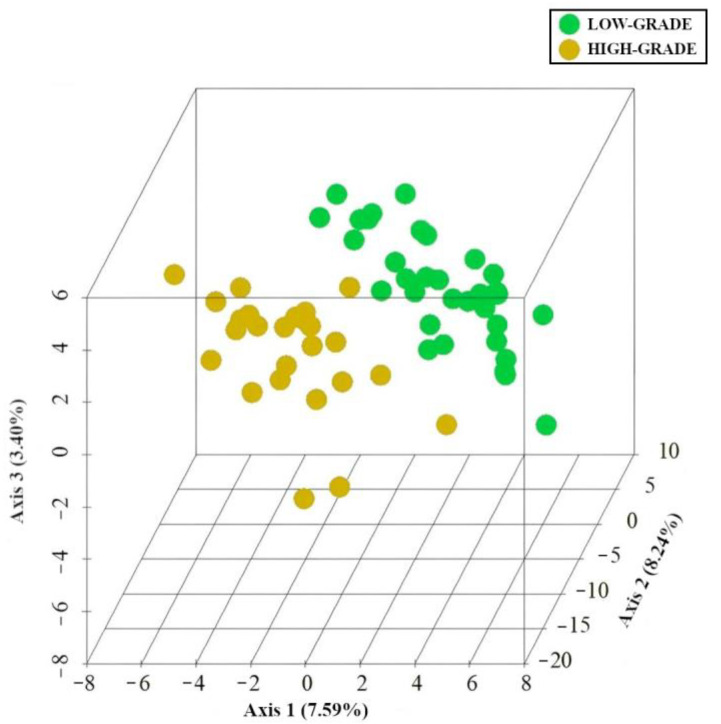
PLS-DA. Specific MAM and mucosa-associated metabolite signatures. PLS-DA analysis of low-grade (green) and high-grade dysplasia (yellow). The three-dimensional score plot indicates the degree of group differences using the first three principal components. Each dot indicates a single sample.

As shown in Figure 4, PLS-DA revealed the presence of specific microbiota and metabolic signatures associated with low- (green) and high-grade dysplasia (yellow).

**Figure 5:**
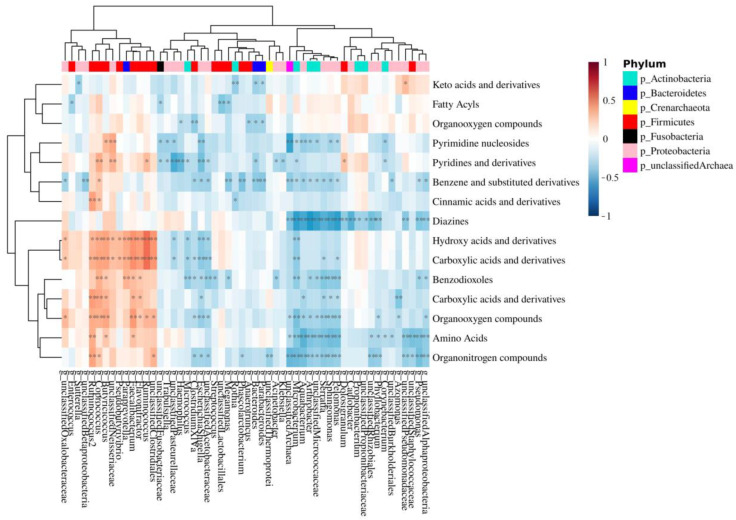
Correlation heat map between MAM genus and mucosa-associated metabolite class. The red color indicates a positive correlation, while the blue color indicates a negative correlation. The darker color indicates the most highly correlated variables. The asterisks indicate the correlation coefficient *p*-value: * *p* < 0.05 and ** *p* < 0.01.

The correlations between modulated genera, significant metabolite classes, and their relative individual metabolites (FC > 1.3 or <0.769, *p* < 0.05) were performed using 16S sequencing and metabolomic data obtained from the analysis of low- and high-grade polyps. Spearman’s rank correlations were calculated between the relative concentration of metabolite classes and the abundance of bacterial taxonomic groups. More than two hundred significant bacteria-metabolite class correlations were identified at the genus level. Fifty-six of these were positive correlations, while 158 were negative correlations (Figure 5). Aromatic compounds were negatively correlated with *Pelomonas, Phascolarctobacterium*, and *Bacteroides,* to which *B. fragilis* belongs (Figure 5). *Pelomonas* and *Phascolarctobacterium* were also negatively correlated with organonitrogen compounds.

## 4. Discussion

In this study, we have analyzed 78 colon polyp patients with the aim of correlating the composition of their gut microbiota and associated metabolome with tumor development. To this end, we devised a novel sampling strategy that enables the collection of mucosa-associated microbiota and metabolome without jeopardizing tumor integrity. By integrating these data, we identified bacteria and metabolites involved in colorectal cancer in a tumor-stage-specific manner.

It is well known that the intestinal microbiota may compromise the mucosal barrier, cross the epithelium, and interact with immune cells, causing local inflammation, cancer induction and progression [2]. Thus, colon cancer microbiota has been generally characterized by using samples collected during surgery to look for bacteria that infiltrate the tumor and shape its microenvironment. We reasoned that this approach would identify mostly passenger bacteria, including not only bacteria with a role in cancer progression, but also those that simply thrive in the cancer microenvironment. Because we wanted instead to identify driver bacteria, we decided to focus on colon adenomas, i.e., benign tumors that have just began the adenoma–carcinoma sequence. Since adenomas are usually excised at colonoscopy to undergo the necessary histological analyses and diagnostic procedures, we devised a strategy that preserved tumor integrity. Our aim was, thus, to investigate whether the surface of intestinal adenomas hosts bacteria that influence cell transformation, given that the bacterial species and metabolites in contact with enterocytes may play an important role in colon carcinogenesis [42,43]. A drawback of this approach is that we cannot compare our samples with healthy neighboring mucosa, since the healthy mucosa is not removed during colonoscopy and therefore is not available for the collection of microbiota and metabolites by brushing.

By comparing the composition of MAM with that of LAM—the former obtained from swabs brushed against the polyp surface, while the latter isolated from fecal samples—we show that the α-diversity indexes (i.e., observed, Shannon, and Simpson) of LAM are significantly higher than those of MAM (Figure 1a–c), and that LAM displays tighter clustering compared to MAM (Figure 1d), in good agreement with previous studies on biopsies from healthy individuals [22,24,44,45].

Despite having higher diversity levels, LAM appears to be more homogeneous than MAM, as shown in Figure 1d (*p* < 0.001). Indeed, LAM displays enrichment of the phyla Firmicutes (Bacillota), Bacteroidetes (Bacteroidota), and Verrucomicrobia, while MAM mainly consists of Proteobacteria (Pseudomonadota) and Actinobacteria (Actinomycetota) (Figure S1). Overall, we found 49 genera that were increased in LAM or MAM samples (*q* < 0.05) (Table S4), consistent with our data at the phylum level. Our findings are also in good agreement with a study by Tang and colleagues [45] showing that, among individuals without gastrointestinal symptoms undergoing routine screening colonoscopies, the phyla Firmicutes (Bacillota) and Bacteroidetes (Bacteroidota) were enriched in LAM, whereas Proteobacteria (Pseudomonadota) were more abundant in biopsy samples. Moreover, analyzing healthy subjects, Ringel and colleagues found that LAM was enriched with Firmicutes (Bacillota)—in agreement with our results and those of Eckburg et al. [23]—and Actinobacteria (Actinomycetota) and less populated by Bacteroidetes (Bacteroidota) and Proteobacteria (Pseudomonadota)—consistent with our results—compared to MAM [22]. In agreement with our data, the mucosal samples analyzed by Sun and colleagues showed an enrichment of *Propionibacterium* (phylum Actinobacteria) and *Escherichia* (phylum Proteobacteria) compared to stool samples [24]. The discrepancy in abundance of some phyla between these literature data and our results can be explained by the fact that our analyses were performed on patients with low- or high-grade dysplastic colorectal polyps, whereas the published data were on healthy subjects. Moreover, differences in experimental procedures such as sampling and extraction protocols, or data analysis could have influenced the results [24,45]. It is also possible that our swab-brushing procedure may lead to discrepancy in MAM composition detection compared to the commonly used biopsies.

We have then characterized the composition of MAM and associated metabolome in colon polyp patients stratified according to their tumor histology (high- vs. low-grade dysplasia). We show that MAM from the low-grade dysplasia group has a larger number of *Pelomonas* and *Phascolarctobacterium* than that of patients with high-grade dysplastic polyps (Figure 2a). Interestingly, *Pelomonas* is associated with the onset of multifocal atrophic gastritis and intestinal metaplasia, well established premalignant gastro-intestinal lesions [46], and is enriched in LAM of CRC patients receiving chemotherapy and/or radiotherapy treatment [47], while *Phascolarctobacterium* is enriched in stool samples of patients with CRC compared to healthy subjects [48]. Overall, our results show that *Pelomonas* and *Phascolarctobacterium* are more abundant in low-grade vs. high-grade dysplasia, which supports the hypothesis that these microorganisms may function as driver bacteria during CRC pathogenesis; however, functional experiments are needed to support this possibility.

At the species level, we detected enrichment of *Bacteroides fragilis* and *Bacteroides* spp. on the surface of low-grade polyps compared to high-grade adenomas (Figure 2b). Thus, it is tempting to speculate that these species may play a role in CRC tumor initiation. Intriguingly, enterotoxigenic *B*. *fragilis* is a well-known driver of CRC [3] due to its oncogenic properties. Among the species found enriched in the low-grade dysplasia group, we also found *unclassified Erysipelotrichaceae incertae sedis*, a species that belongs to the *Erysipelotrichaceae* family. Interestingly, the levels of intestinal *Erysipelotrichaceae* are reduced in the LAM of patients with advanced colon adenomas compared to healthy subjects [49], and increased in hyperplastic polyps compared to adenocarcinomas [47]. Lastly, bacteria belonging to this family play an important role in inflammation [50] and are associated with increased levels of inflammatory markers involved in tumor growth, invasion, and metastasis [51]. The lack of *Fusobacteria* enrichment in our samples can be explained by the fact that *Fusobacterium* spp. colonize more advanced CRC tissues [52].

With regard to the metabolome, we succeeded in identifying 41 metabolites differentially associated with low- and high-grade dysplasia groups, with 29 of them previously found enriched in LAM of CRC patients compared to healthy subjects [48,53,54,55,56,57,58]. These findings confirm and extend our previous work on tumor-associated metabolites isolated from 20 patients [31]. More specifically, we found erythronic acid (FC = 0.7) (Figure S4) enriched in low- compared to high-grade dysplastic polyps, a metabolite produced by bacteria from the Actinobacteria (Actinomycetota) and Proteobacteria (Pseudomonadota) phyla [56]. Fittingly, we found enrichment of the aforementioned genus *Pelomonas*, which belongs to Proteobacteria (Pseudomonadota), in low-grade dysplastic polyps.

In contrast, microbiota analyses of high-grade dysplastic adenomas showed an enrichment of the genus *Anaerococcus* (Figure 2b), that was found significantly enriched in CRC tissues and is considered a potential passenger genus [4]. Further functional studies are needed to evaluate the role of *Anaerococcus* in cancer progression.

As expected, our results indicate a gradual replacement of the potential driver bacteria by the potential passenger bacteria in high-grade adenomas. These microorganisms are opportunistic pathogens—possibly involved in CRC progression—taking advantage of the changes occurring in the TME to colonize it even further. In fact, we did not identify any enrichment of potential passenger genera or species in low-grade dysplastic samples. In this regard, a limitation of the present work is that we cannot classify bacteria as driver or passenger based on our results, but rather must rely on previously suggested classifications, which, however, are often based on disease–bacteria correlations and not on functional experiments at a mechanistic level.

Upon analysis of the polyp-associated metabolome, we found L-serine (FC = 2.8) and threonine (FC = 8.2) enriched in high- vs. low-grade dysplastic polyps (Figure S4). Interestingly, Garza et al. have recently published a computational model explaining the association between passenger bacteria and CRC metabolites, such as L-serine and threonine [17]. These authors suggest that changes in metabolite composition may allow opportunistic passenger bacteria to colonize tumor sites [17]. Moreover, a more recent study has shown that L-serine is required for CRC cell proliferation, and that dysregulation of serine metabolism is closely related to the occurrence and development of this tumor [59]. Two other metabolites found enriched in the high-grade dysplasia group were lactic acid (FC = 1.9) and butyric acid (FC = 3.7) (Figure S4), which may both derive from the aerobic glycolysis occurring in CRC cells and/or the metabolism of gut bacteria. In fact, cancer cells are able to perform aerobic glycolysis even under oxygen availability, thereby producing lactate from pyruvate [60,61], a phenomenon known as the Warburg effect [62]. Due to this metabolic switch to aerobic glycolysis, cancer cells are unable to efficiently metabolize butyrate, the primary energy source of normal colonocytes. On the other hand, as aforementioned, our samples from the high-grade dysplasia group displayed increased levels of *Anaerococcus* spp. (Figure 2b), which belongs to the phylum Firmicutes (Bacillota) known to be a major source of butyric acid and lactic acid [63].

PICRUSt analysis on MAM identified four pathways statistically enriched in low-grade and 13 in high-grade dysplasia groups. Interestingly, the high-grade group showed an enrichment of the mixed acid fermentation pathway and superpathway of pyrimidine ribonucleosides salvage. The first one leads to the production of lactic acid, while the second one of uracil, both metabolites enriched in high-grade dysplastic polyps (Figures S4 and S7). It is worth noting that the PICRUSt analysis provides only a prediction of the functions of the bacterial community.

The integration of bacterial genera and metabolite class data, crucial to understanding the relationship between bacterial genera and metabolite classes [64], shows that the two genera, *Pelomonas* and *Phascolarctobacterium*, enriched in the low-grade dysplasia group, are negatively correlated with organonitrogen compounds (Figure 5). Interestingly, betaine, carnitine, and choline, whose high levels in the plasma of human subjects have been shown to reduce CRC risk [65], are organonitrogen compounds. In particular, they all are trimethylamine (TMA) precursors that can be metabolized to trimethylamine-N-oxide (TMAO) by *Phascolarctobacterium* [66]. TMAO is known to promote CRC progression not only through N-Nitroso compounds formation [67], which leads to DNA damage, but also via the production of reactive oxygen species (ROS) [68]. Thus, it is possible that an increase in *Pelomonas* and *Phascolarctobacterium*, as demonstrated by the present study, may ultimately lead to the downregulation of betaine, carnitine, and choline as the latter may be used by the bacteria to produce TMA and consequently, TMAO. Unfortunately, the method used for the analysis of mucosal-adherent metabolome is not suitable for the quantification of TMAO, so further analyses are needed to confirm this hypothesis.

Lastly, the genera, *Pelomonas*, *Phascolarctobacterium*, and *Bacteroides*, the latter of which comprises the species *B. fragilis*, (Figure 5), showed a negative correlation with benzene and benzene derivatives, which include benzoic acid and its substituted derivatives endowed with HDAC inhibitory activity [69]. Thus, it is conceivable that the decrease in benzene and derivatives may be involved in CRC development.

## 5. Conclusions

**Figure 6:**
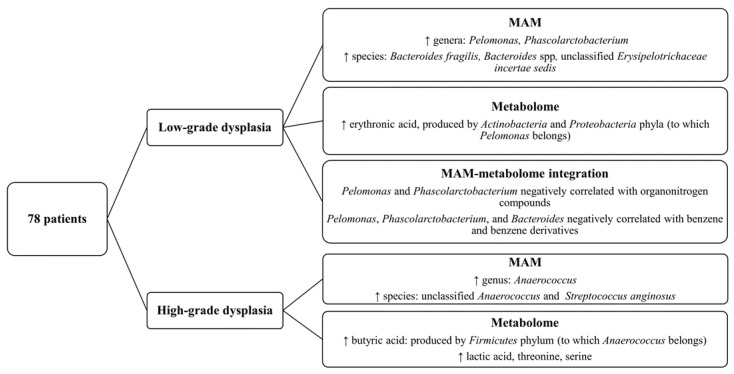
Diagram showing the key findings of this article about MAM and mucosa-associated metabolome of patients with high- or low-grade dysplastic polyps.

In conclusion, our findings (summarized in Figure 6), based on a novel sampling strategy, support the hypothesis of a direct and indirect involvement of the gut microbiota and their metabolites in CRC initiation and early progression, since we found different signatures in low-grade dysplastic polyps compared with high-grade ones, which represent more advanced stages of the adenoma–carcinoma sequence. Our results also stress the importance of analyzing tumor-associated microbiota and metabolome to identify key carcinogenic pathogens, whereas luminal-derived data, although still essential as clinical markers, only seem to recapitulate the general gut environment.

## Figures and Tables

**Table 1:** Clinical features of the patients. Statistically significant *p*-values (*p* < 0.05) are in bold.

Clinical Features	Patients n = 78 (%)	Patients with Low-Grade Dysplastic Polyps n = 44 (%)	Patients with High-Grade Dysplastic Polyps n = 34 (%)	*p*-Value
**Gender**				
Female	33 (42.3%)	21 (47.7%)	12 (35.3%)	0.3
Male	45 (57.7%)	23 (52.3%)	22 (64.7%)	
**BMI (body mass index)**				
Normal weight	34 (43.6%)	20 (45.4%)	14 (41.2%)	0.7
Overweight or obese	44 (56.4%)	24 (54.6%)	20 (58.8%)	
**Age**				
Median (IQR)	61 (58–70)	61 (58–68)	62 (56–70)	0.9
**Polyp size mm**				
Median (IQR)	14 (10–23)	12 (10–16)	15 (12–25)	**0.02**
**Type of polyp**				
Tubular	28 (35.9%)	21 (47.7%)	7 (20.6%)	**0.005**
Villous	3 (3.8%)	1 (2.3%)	2 (5.9%)	
Tubulo-villous	40 (51.3%)	16 (36.4%)	24 (70.6%)	
Others	7 (9.0%)	6 (13.6%)	1 (2.9%)	
**Previous gastrointestinal conditions**				
Diverticulitis	26 (33.3%)	19 (43.2%)	7 (20.6%)	0.2
Previous polyp occurrence	8 (10.2%)	6 (13.6%)	2 (5.9%)	
IBD	1 (1.3%)	0	1 (2.9%)	
Previous cholecystectomy	5 (6.4%)	4 (9.1%)	1 (2.9%)	
Slight mucosal inflammation	1 (1.3%)	0	1 (2.9%)	
**Polyp localization**				
Right colon	18 (23.1%)	11 (25.0%)	7 (20.6%)	0.8
Left colon	52 (66.7%)	28 (63.6%)	24 (70.6%)	
Transversal colon	8 (10.2%)	5 (11.4%)	3 (8.8%)	

BMI: body mass index; IQR: interquartile range; IBD: inflammatory bowel disease.

**Table 2:** Pathways differently enriched in patients with low- (green) or high-grade (yellow) dysplastic polyps, obtained by PICRUSt analysis. * Pathway described in article Discussion.

Pathway	Description	*p*-Value
Superpathway of adenosylcobalamin salvage from cobinamide I (COBALSYN-PWY)	Vitamin biosynthesis	8.54 × 10^−3^
Adenosylcobalamin biosynthesis from adenosylcobinamide-GDP I (PWY-5509)	Vitamin biosynthesis	0.016
Superpathway of adenosylcobalamin salvage from cobinamide II (PWY-6269)	Vitamin biosynthesis	2.78 × 10^−3^
Sucrose degradation IV (sucrose phosphorylase) (PWY-5384)	Carbohydrate degradation	0.035
Mixed acid fermentation (FERMENTATION-PWY) *	Carbohydrate degradation	5.94 × 10^−3^
Superpathway of tetrahydrofolate biosynthesis and salvage (FOLSYN-PWY)	Vitamin biosynthesis	0.036
Superpathway of tetrahydrofolate biosynthesis (PWY-6612)	Vitamin biosynthesis	0.034
Superpathway of thiamine diphosphate biosynthesis II (PWY-6895)	Vitamin biosynthesis	0.034
Superpathway of purine nucleotides de novo biosynthesis II (DENOVOPURINE2-PWY)	Nucleotides synthesis	6.73 × 10^−3^
Superpathway of guanosine nucleotides de novo biosynthesis II (PWY-6125)	Nucleotides synthesis	0.028
Superpathway of pyrimidine ribonucleosides salvage (PWY-7196) *	Nucleotides synthesis	7.02 × 10^−3^
Superpathway of guanosine nucleotides de novo biosynthesis I (PWY-7228)	Nucleotides synthesis	0.036
Superpathway of purine nucleotides de novo biosynthesis I (PWY-841)	Nucleotides synthesis	0.038
Superpathway of pyrimidine ribonucleotides de novo biosynthesis (PWY0-162)	Nucleotides synthesis	0.031
Incomplete reductive TCA cycle (P42-PWY)	Reductive TCA cycle	0.017
PreQ0 biosynthesis (PWY-6703)	Secondary metabolite biosynthesis	0.049
Pyrimidine deoxyribonucleotides de novo biosynthesis II (PWY-7187)	Nucleoside and nucleotide synthesis	0.019

## Data Availability

The dataset comprising raw 16S rRNA sequences generated during the current study was deposited in the National Center for Biotechnology Information (NCBI) database. BioProject MIMEC Project_Swab PRJNA783496 (https://submit.ncbi.nlm.nih.gov/subs/sra/SUB10703567/overview, accessed on 17 December 2021) and MIMEC Project_Fecal PRJNA783535 (https://submit.ncbi.nlm.nih.gov/subs/sra/SUB10721767/overview, accessed on 17 December 2021).

## Supplementary Materials

The following supporting information can be downloaded at: https://www.mdpi.com/article/10.3390/cancers15123065/s1, Table S1: Previous gastrointestinal conditions reported by the analyzed patients with low- (green) or high-grade (yellow) dysplastic polyps; Table S2: comparison of nutrient intake between patients with low- and high-grade dysplastic polyps; Table S3: metabolomics data; Table S4: Genera that distinguish MAM and LAM. Negative LDA score: enrichment in MAM. Positive LDA score: enrichment in LAM. Only statistically significant genera were included in the table (*p*-value and FDR < 0.05). The updated names covered by the International Code of Nomenclature for Prokaryotes are indicated in parentheses ([70]); Figure S1: pie chart and linear discriminant analysis effect size (LDA-LEfSe) of the comparison between MAM and LAM; Table S4: genera that distinguish MAM and LAM; Figure S2: phylogenetic heat tree showing differences in the relative abundance of MAM taxa between high-grade dysplastic (yellow) vs. low-grade dysplastic polyps (green); Figure S3: linear discriminant analysis effect size (LDA-LEfSe) showing (a) bacterial genera and (b) bacterial species enriched in LAM high- (yellow; LDS score > 2) vs. low-grade dysplastic adenomas (green; LDA score < −2); Figure S4: box plots of the most significant molecules discriminating low- (green) from high-grade (yellow) dysplastic adenomatous polyps; Figure S5: volcano plot of quantified metabolites; Figure S6: alternative representation of the hierarchical clustering heat-map reported in Figure 3a. Here, the patients with low-grade dysplastic polyps (green) are grouped on the right, while the patients with high-grade dysplastic polyps (yellow) are grouped on the left; Figure S7: products of mixed acid fermentation pathway (a) and superpathway of pyrimidine ribonucleosides salvage (b).
