# Supplementary material for: Distinct Signatures of Tumor-Associated Microbiota and Metabolome in Low-Grade vs. High-Grade Dysplastic Colon Polyps: Inference of Their Role in Tumor Initiation and Progression
Source: Cancers (Basel). 2023 Jun 6;15(12):3065. doi: 10.3390/cancers15123065 (PMC10296265; doi:10.3390/cancers15123065)
Supplement: Supplementary file 1 [file cancers-15-03065-s001.zip › Supplementary Material.pdf]

Supplementary Material

Low-grade	Previous gastrointestinal diseases or procedures	High-grade	Previous gastrointestinal diseases or procedures
8	No	7	Diverticulitis
16	Diverticulitis	10	Diverticulitis
18	Previous polyps occurrence	11	Diverticulitis
20	Diverticulitis	12	No
21	Diverticulitis; previous polyps occurrence	15	No
22	No	28	No
23	No	31	No
25	No	36	No
26	No	44	No
29	No	48	No
32	No	52	No
34	No	53	No
37	Diverticulitis	54	Cholecystectomy (65 years)
39	Cholecystectomy (57 years)	72	IBD at diagnosis, no treatment before colonoscopy and LAM collection
47	Diverticulitis	86	No
49	Previous polyps occurrence	97	No
50	No	99	No
51	No	105	No
57	Diverticulitis	110	Diverticulitis
58	Diverticulitis	112	Diverticulitis
59	Diverticulitis	119	No
60	Previous polyps occurrence	120	No
64	No	121	Previous polyps occurrence
65	No	122	No
66	Previous polyps occurrence	123	No
68	No	124	Diverticulitis; previous polyps occurrence
69	Diverticulitis	128	Diverticulitis
71	Diverticulitis	138	No
73	Diverticulitis	140	Slight mucosal inflammation
77	Diverticulitis	141	No
83	No	155	Diverticulitis
107	Diverticulitis	156	No
111	Diverticulitis; previous polyps occurrence	160	No
113	Cholecystectomy (79 years); diverticulitis	162	No
125	Cholecystectomy (73 years); diverticulitis		
130	Cholecystectomy (55 years)		
132	Diverticulitis		
137	No		
139	Previous polyps occurrence		
153	No		
154	Diverticulitis		
157	No		
159	Diverticulitis		
163	No		

Table S1. Previous gastrointestinal conditions reported by the analyzed patients with low- (green) or high-grade (yellow) dysplastic polyps.

Nutrient	Median g/day (IQR)	*p*-value
**Red meat**		
Low-grade	29.4 (15.7-60.4)	0.5
High-grade	42.6 (23.2-58.4)	
**Red and processed meat**		
Low-grade	49.5 (29.6-90.9)	0.4
High-grade	65.5 (45.6-84.6)	
**Fruit and vegetables**		
Low-grade	375.8 (328.1-525.4)	0.8
High grade	431.3 (292.3-560.3)	
**Fibers**		
Low-grade	18.2 (13.4-21.8)	0.6
High grade	19.5 (12.6-23.4)	
**Lipids**		
Low-grade	71.6 (47.5-85.6)	0.5
High-grade	73.8 (56.3-90.2)	

Table S2. Comparison of nutrient intake between patients with low- and high-grade dysplastic polyps. Table S2 shows the median value for the two groups and the interquartile range (IQR). The *p*-value was obtained using Mann-Whitney statistical test. A p-value < 0.05 was considered statistically significant.

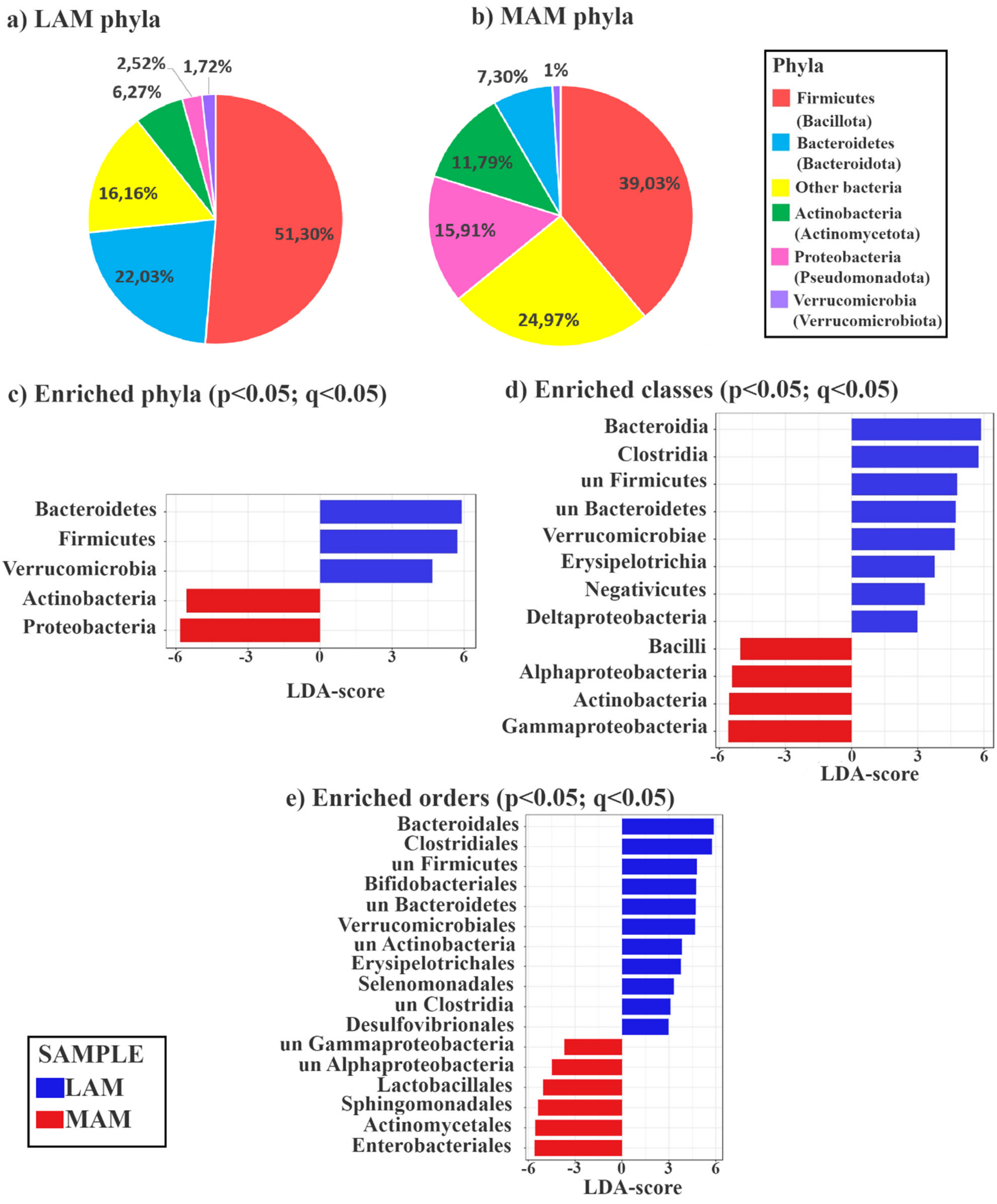

Fig. S1. Pie chart showing the relative abundance percentage of different phyla in LAM (a) *vs* MAM (b). LefSe representing phyla (c) classes (d) and orders (e) significantly enriched in LAM (blue) or MAM (red).

Phylum	***Genus***	***Pvalues***	***FDR***	***LDAscore***
Actinobacteria (Actinomycetota)	*Propionibacterium*	7,42E-24	4,75E-22	-4.89
Actinobacteria (Actinomycetota)	*unclassified_Actinomycetales*	2,38E-20	7,19E-18	-4.14
Actinobacteria (Actinomycetota)	*Arthrobacter*	3,30E-13	2,35E-12	-5.14
Actinobacteria (Actinomycetota)	*unclassified_Micrococcaceae*	4,94E-13	3,16E-12	-5.14
Actinobacteria (Actinomycetota)	*unclassified_Bifidobacteriaceae*	1,40E-01	2,89E-01	4.39
Actinobacteria (Actinomycetota)	*unclassified_Coriobacteriaceae*	0.0043271	0.0067545	-2.89
Actinobacteria (Actinomycetota)	*unclassified_Actinobacteria*	0.0084067	0.01281	3.84
Bacteroidetes (Bacteroidota)	*unclassified_Bacteroidales*	3,37E-19	7,19E-18	5.19
Bacteroidetes (Bacteroidota)	*unclassified_Bacteroidetes*	9,98E-18	1,38E-16	4.72
Bacteroidetes (Bacteroidota)	*unclassified_Porphyromonadaceae*	1,58E-16	1,68E-15	4.32
Bacteroidetes (Bacteroidota)	*unclassified_Rikenellaceae*	2,03E-12	1,09E-11	3.54
Bacteroidetes (Bacteroidota)	*Barnesiella*	1.43e-13	6,54E-09	4.75
Bacteroidetes (Bacteroidota)	*Bacteroides*	5,47E-09	2,34E-08	5.51
Bacteroidetes (Bacteroidota)	*Alistipes*	2,55E-08	1,02E-08	4.31
Bacteroidetes (Bacteroidota)	*Parabacteroides*	4,10E-09	1,54E-08	4.89
Bacteroidetes (Bacteroidota)	*Odoribacter*	2,65E-06	8,49E-06	3.66
Bacteroidetes (Bacteroidota)	*Butyricimonas*	3,93E-05	1,20E-04	3.64
Bacteroidetes (Bacteroidota)	*unclassified_Prevotellaceae*	5,41E-02	1,24E-01	4.81
Bacteroidetes (Bacteroidota)	*Prevotella*	0.017516	0.023851	4.04
Firmicutes (Bacillota)	*unclassified_Clostridiales*	2,11E-14	1,93E-13	5.19
Firmicutes (Bacillota)	*Faecalibacterium*	5,93E-11	2,92E-10	5.44
Firmicutes (Bacillota)	*unclassified_Firmicutes*	1,90E-07	6,77E-07	4.79
Firmicutes (Bacillota)	*Roseburia*	2,90E-07	9,76E-07	4.52
Firmicutes (Bacillota)	*Flavonifractor*	5,67E-05	1,65E-04	3.54
Firmicutes (Bacillota)	*Oscillibacter*	1,60E-04	4,46E-05	3.76
Firmicutes (Bacillota)	*Lachnospiracea_incertae_sedis*	1,41E-03	3,77E-03	4.76
Firmicutes	*unclassified_Lachnospiraceae*	2,94E-02	7,23E-02	5.31

(Bacillota)				
Firmicutes (Bacillota)	*unclassified_Peptostreptococcaceae*	4,27E-02	1,01E-01	3.93
Firmicutes (Bacillota)	*Ruminococcus*	8,30E-03	1,83E-01	4.54
Firmicutes (Bacillota)	*unclassified_Clostridia*	9,47E-02	2,02E-01	3.1
Firmicutes (Bacillota)	*Streptococcus*	3,53E-01	7,06E-01	-4.98
Firmicutes (Bacillota)	*Butyricicoccus*	7,33E-01	0.00014221	2.84
Firmicutes (Bacillota)	*Clostridium_XlVa*	0.00034832	0.00064562	3.87
Firmicutes (Bacillota)	*unclassified_Erysipelotrichaceae*	0.00035307	0.00064562	4.15
Firmicutes (Bacillota)	*unclassified_Ruminococcaceae*	0.00073058	0.0012637	4.65
Firmicutes (Bacillota)	*unclassified_Selenomonadales*	0.0034355	0.0056377	3.32
Firmicutes (Bacillota)	*Clostridium_XlVb*	0.0036883	0.0059012	-3.08
Firmicutes (Bacillota)	*unclassified_Streptococcaceae*	0.010488	0.015255	-3.79
Firmicutes (Bacillota)	*unclassified_Veillonellaceae*	0.012696	0.018057	3.12
Firmicutes (Bacillota)	*Clostridium_XI*	0.015786	0.021963	3.66
Proteobacteria (Pseudomonadota)	*unclassified_Sphingomonadaceae*	1,08E-17	1,38E-16	-4.4
Proteobacteria (Pseudomonadota)	*unclassified_Alphaproteobacteria*	1,10E-13	8,76E-13	-4.49
Proteobacteria (Pseudomonadota)	*Sphingomonas*	8,49E-13	4,94E-12	-5.33
Proteobacteria (Pseudomonadota)	*unclassified_Gammaproteobacteria*	4,08E-03	1,04E-02	-3.68
Proteobacteria (Pseudomonadota)	*Bilophila*	0.00059991	0.0010665	2.98
Proteobacteria (Pseudomonadota)	*Escherichia_Shigella*	0.00081832	0.0013782	-5.39
Proteobacteria (Pseudomonadota)	*Gemmiger*	0.019303	0.025737	-3.73
Proteobacteria (Pseudomonadota)	*unclassified_Enterobacteriaceae*	0.026979	0.035238	-5.18
Verrucomicrobia (Verrucomicrobiota)	*Akkermansia*	0.010152	0.015109	4.68

Table S4. Genera that distinguish MAM and LAM. Negative LDA score: enrichment in MAM. Positive LDA score: enrichment in LAM. Only statistically significant genera were included in the table (*p*-value and FDR < 0.05). The updated names covered by the International Code of Nomenclature for Prokaryotes are indicated in parentheses (Oren A, Garrity GM. Valid publication of the names of forty-two phyla of prokaryotes. Int J Syst Evol Microbiol. 2021 Oct;71(10). doi: 10.1099/ijsem.0.005056)

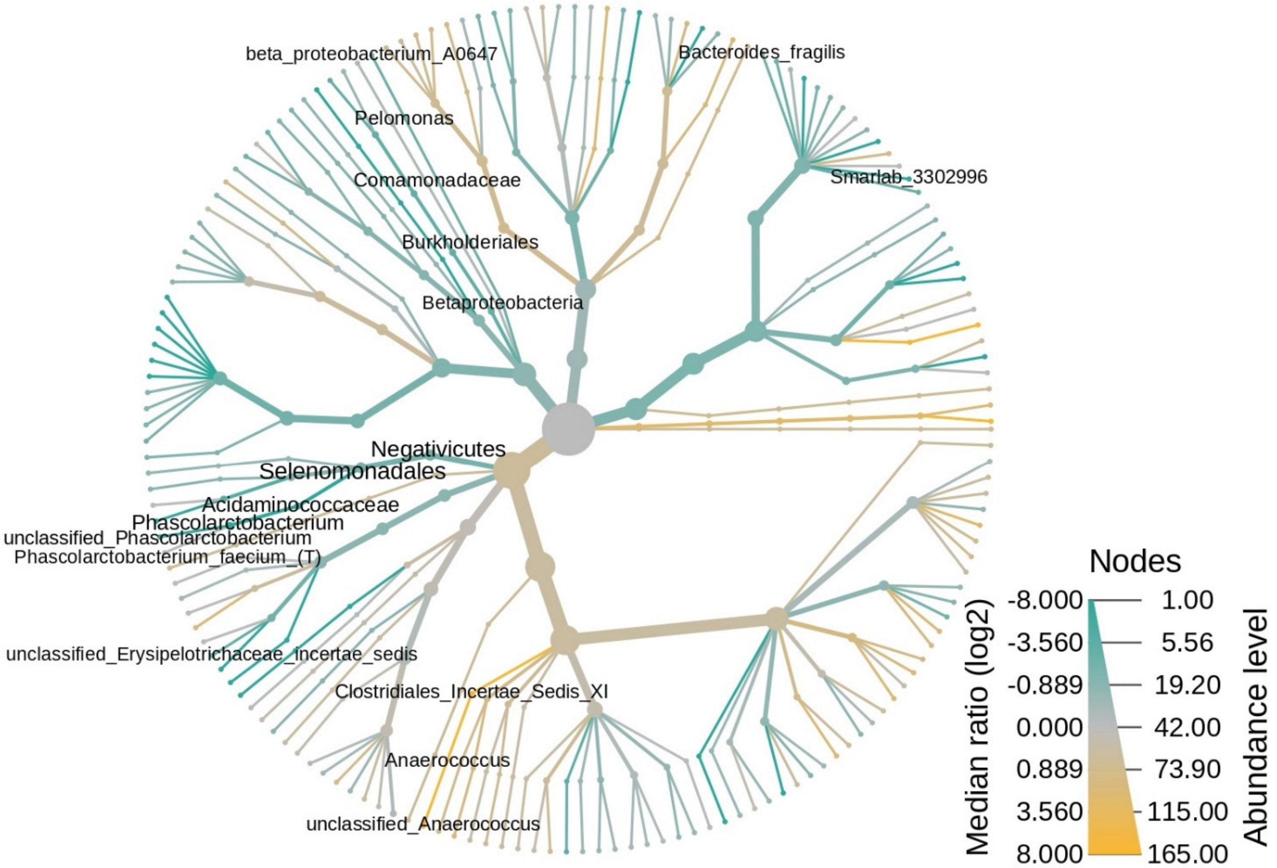

Fig. S2. Phylogenetic heat tree showing differences in the relative abundance of MAM taxa between low- (green) and high-grade dysplasia (yellow) groups. Phyla, classes, orders, families, genera, and species are represented. The nodes indicate the hierarchical structure of taxa. Only the nodes with a statistically significant difference ($p < 0.05$) between the two groups are labeled.

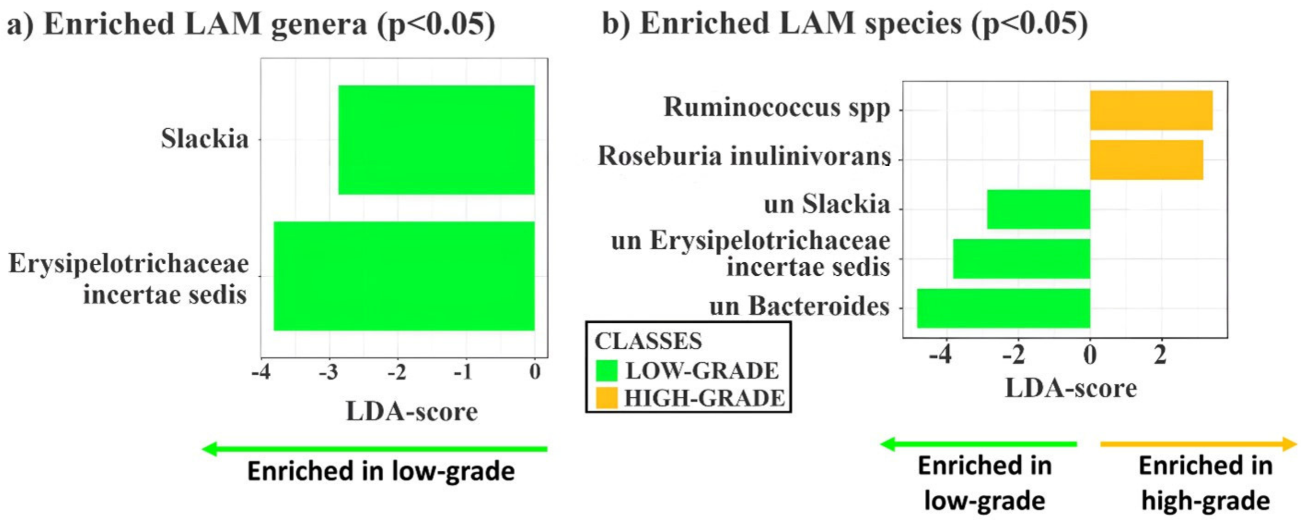

Fig. S3. Linear discriminant analysis effect size (LDA-LEfSe) showing (a) bacterial genera and (b) species enriched in LAM high-grade dysplastic (yellow; LDA score > 2) *vs* low-grade dysplastic polyps (green; LDA score < -2). This method incorporates statistical significance (Kruskal-Wallis) with biological consistency (effect size). The length of the bar represents a log10 transformed LDA score. This value is positive if the bacterial species is enriched in the first compared to the second group and negative if the second group shows enrichment compared to the first group. A significance level of $p < 0.05$ and an LDA score of 2 and -2 are used to determine the species best characterizing each phenotype.

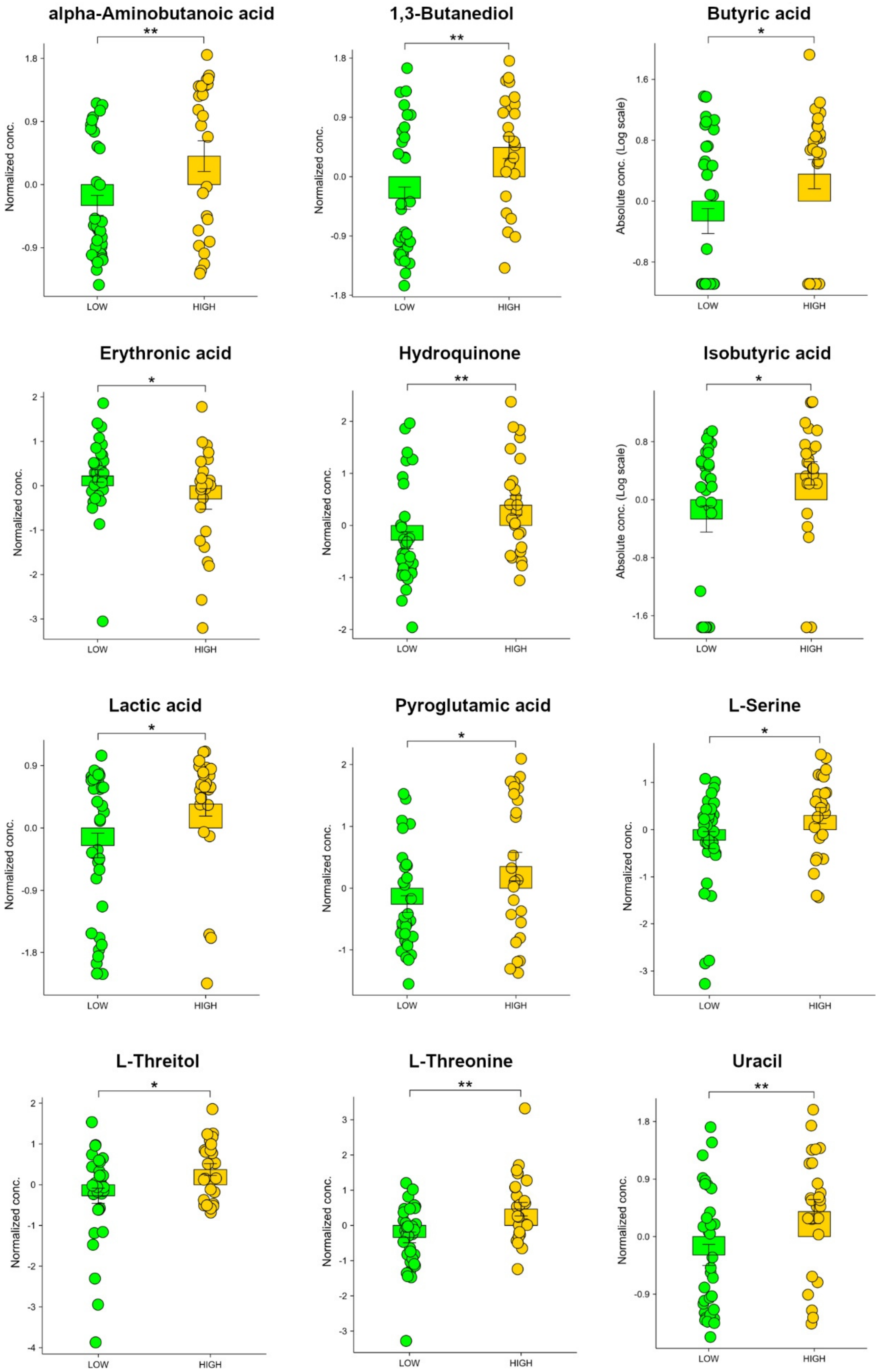

Fig. S4. Box plots of the most statistically significant molecules discriminating low- (green) from high-grade (yellow) dysplastic adenomatous polyps (**p* < 0.05; ***p* < 0.01).

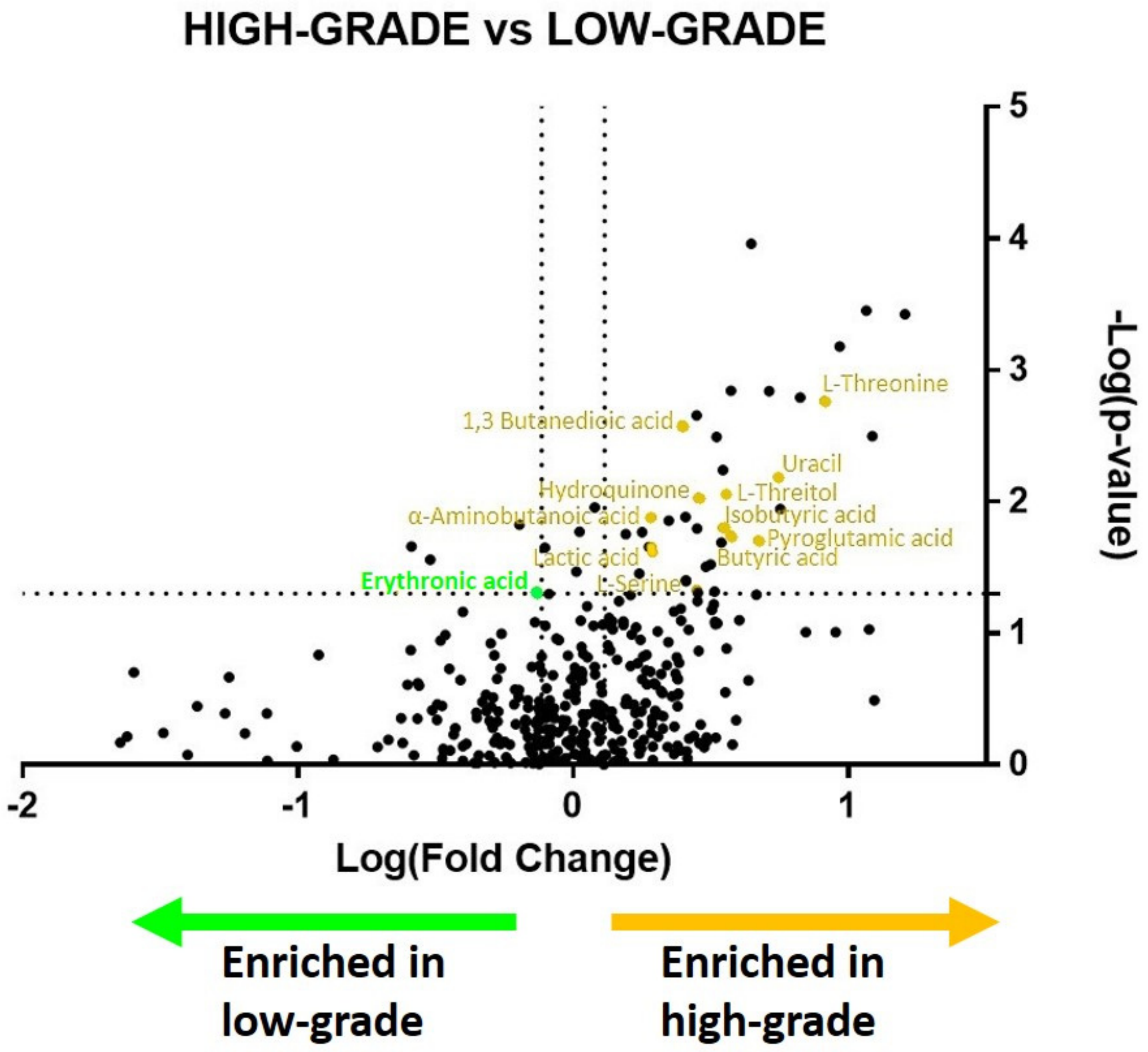

Fig. S5. Volcano plot of quantified metabolites. The figure shows the metabolites enriched in low- (green; on the left) or high- (yellow; on the right) grade dysplastic polyps that are discussed in the text.

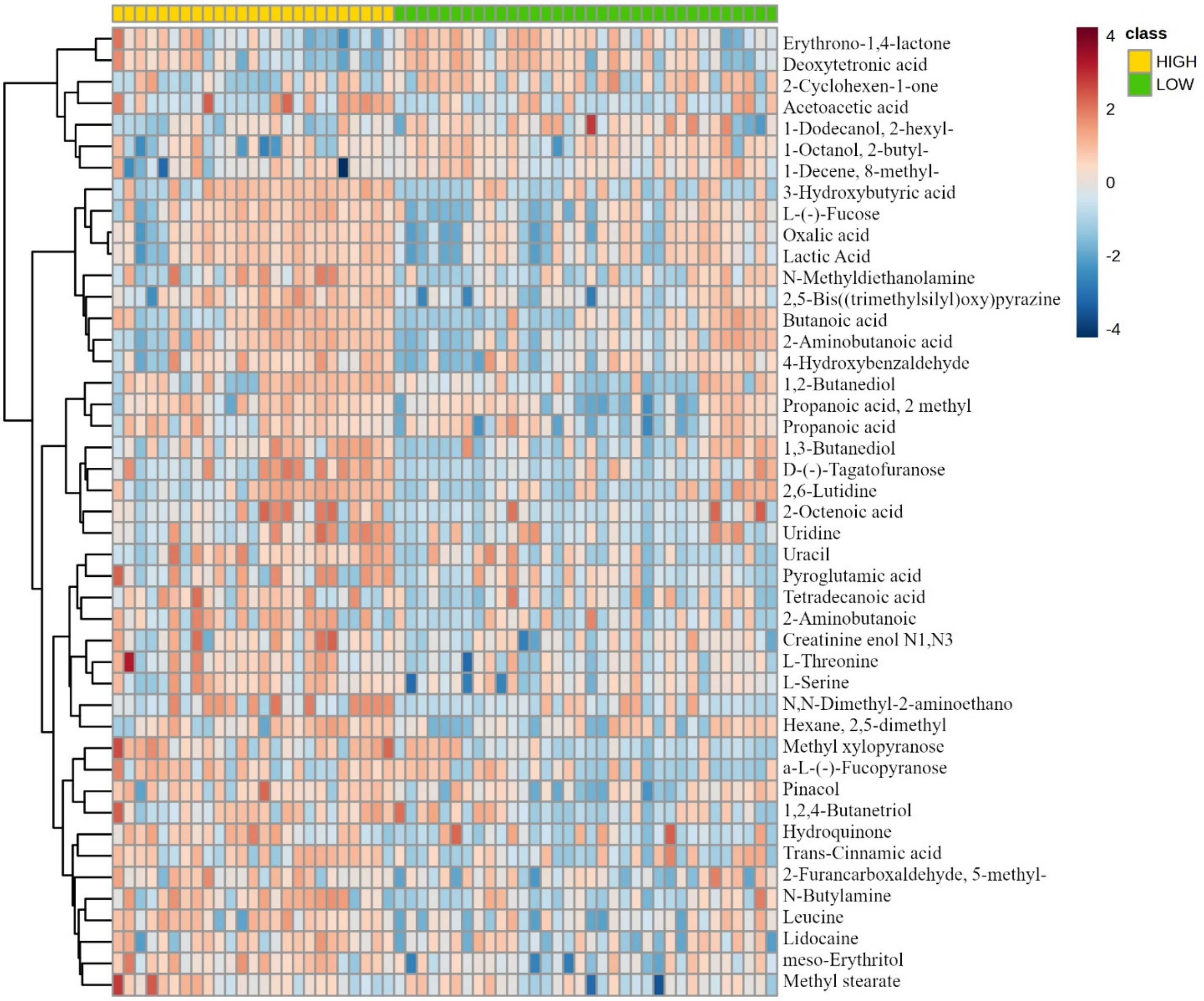

Fig. S6. Alternative representation of the hierarchical clustering heat-map reported in Figure 3a. Here the patients with low-grade dysplastic polyps (green) are grouped on the right, while the patients with high-grade dysplastic polyps (yellow) on the left.

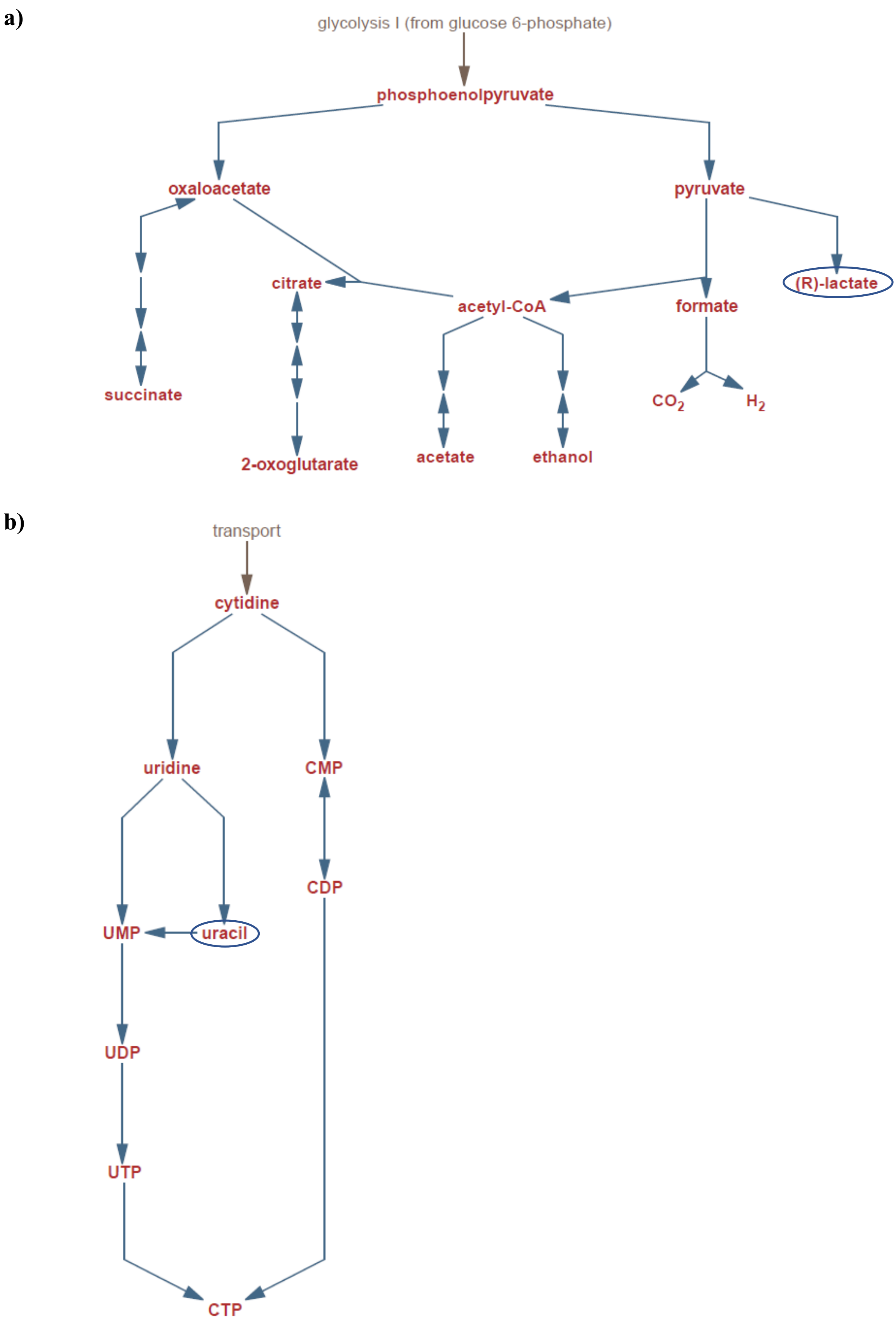

Fig. S7. Products of mixed acid fermentation pathway (a), and superpathway of pyrimidine ribonucleosides salvage (b) from the MetaCyc website (https://metacyc.org/). Circled products are discussed in the text.
